# Parents’ Knowledge, Attitude, and Practice Regarding Traditional Medicine on Their Children: A Community-Based Cross-Sectional Study in Bisha City, Saudi Arabia

**DOI:** 10.7759/cureus.43136

**Published:** 2023-08-08

**Authors:** Jaber A Alfaifi, Saad Ali M Alqarni, Anas Alqarni, Masoud M Alqahtani, Raydaa A Alshomrani

**Affiliations:** 1 Pediatrics, College of Medicine, University of Bisha, Bisha, SAU; 2 College of Medicine, University of Bisha, Bisha, SAU

**Keywords:** parents, practice, attitude, knowledge, traditional medicine

## Abstract

Background

The use of traditional medicine (TM) in children is widespread, particularly in developing countries. Parents often rely on TM to treat their children's illnesses or maintain their health. However, the safety and efficacy of TM are often unclear, and there is a need to assess parents' knowledge, attitudes, and practices toward its use.

Methods

This is a community-based cross-sectional study conducted in Bisha, Saudi Arabia, with a sample size of 555. The study used a modified questionnaire to collect data. The data were collected from March to June 2023. This study involved both descriptive and inferential statistics.

Results

The study included 555 participants, most of whom were female, Saudi nationals, and married and had a bachelor's degree. More than half of the participants reported using TM, and most believed that it had fewer side effects and could be taken with allopathic medicines. However, many participants did not believe that TM could prevent or cure all diseases or that it was always safe. The median knowledge score was 4.0, with higher scores associated with older age and higher educational level. Most participants had a positive attitude toward TM, with higher attitude scores associated with younger age, male gender, lower educational level, and healthcare-related occupation. The median practice score was 31.0, with higher scores associated with younger age, male gender, illiteracy, and healthcare-related occupation. Overall, the study highlights the importance of understanding the patterns of use, knowledge, attitudes, and practices of TM in the population, particularly among different demographic groups.

Conclusions

This study highlights the need for better regulation and supervision of TM outlets to ensure the safety and efficacy of the products. It also emphasizes the importance of consulting healthcare professionals before using TM on children. The findings suggest that healthcare providers should be knowledgeable about TM and provide guidance to parents on its appropriate use.

## Introduction

Traditional medicine (TM) can be defined as knowledge, theories, and practice used to prevent, diagnose, and treat various diseases. TM represents the most ancient medical profession information passed from generation to generation over centuries. A major part of TM is herbal medicine that is used by 75-80% of the world's population, especially in developing countries, as it is considered safer than synthetic drugs [1]. TM includes prophetic medicine, herbal medicine, cupping, splinting fractures, camel's products, honey and bee products, cauterization, sulfurous mineral waters, compounds mixtures, herbal mixtures, and sand baths.

Recently, there has been a worldwide increase in TM or natural medicine use. Statistics indicate that over 70% of people around the globe have a significant amount of knowledge of TM and use them often [2]. TM existed in human societies long before the application of modern science to health. Many studies have been conducted around the globe on the use of TM, but there are limited studies done on parents’ attitudes and perceptions of their children on the use of the same [3]. Statistics emanating from previous studies show that parents in entire America had at least administered herbal drugs or products to almost three million children [4]. For Germany, a higher number of children was recorded to have used herbal drugs, with only a 14.5% minority having been left out. Globally, it has been evidenced that the source of knowledge on the general practice of TM by parents to their children has greatly been left out of a study by researchers [4]. In addition, laws that govern the use of TM in the Kingdom of Saudi Arabia (KSA) are weak and vastly ineffective [5]. The use of TM has been embraced around the world, with countries such as Ethiopia recording a TM usage rate of 88% by parents on their children. The most common of these TMs are herbs, massages, religious therapies, and traditional midwifery. Various factors have been observed to influence the use of TM in children.

Gender was a key factor, with female parents more likely to use TM on their children. Low levels of education, accessibility of TM, pricing, and the general perceived safety and efficacy of TM are factors that influence the prevalence, perception, and usage of TM [6]. The practice of TM in the KSA as a treatment option by parents for their children is not evenly determined among regions, and different areas have different practice magnitudes depicting this; TM regulations might be non-existence [7]. A score of factors globally has propagated the rise in the practice of TM by parents on children for the last two decades. The easy accessibility of TM has been pointed out as one of the catalysts to the practice, without undermining the fact that most parts of the world, especially Africa and Asia, have negligible access to conventional treatment [1].

Since the beginning of motherhood, mothers have typically relied on herbal restoratives. This has been evidenced by the use of TM to increase breast milk without much knowledge of the health risks it could pose to their infants indirectly, and this shows that parents’ practice of TM has been heavily rooted in childbirth all through parenting [7]. This study aims to assess parents' knowledge, attitude, and practice toward TM in Bisha City, Saudi Arabia, 2023.

## Materials and methods

This research employed a community-based descriptive, cross-sectional study design. The study was conducted in Bisha, a city located in the northern part of the Asir region, approximately 2,000 feet above sea level. As of the 2017 Census, the city had an estimated population of 204,491 [8]. 

The study included parents residing within Bisha City who expressed willingness to participate and provided they had children. Individuals were eligible for inclusion irrespective of their gender, education level, occupation, or nationality. Parents residing outside the boundaries of Bisha City or those without children were excluded from the study.

The sample size was calculated using the Raosoft calculator, which yielded a sample size of 384. Considering potential participation bias, the sample size was augmented by 44% to a total of 555 respondents.

Data collection was performed using a modified version of a questionnaire previously utilized in a study titled "Public Knowledge, Attitudes, and Practices towards Herbal Medicines; A Cross-Sectional Study in Western Saudi Arabia" [9]. The self-administered questionnaire, divided into distinct sections, was designed to gather specific data types. These sections included demographic characteristics, usage and knowledge of TM, and attitudes and practices among respondents. The questionnaire was distributed electronically.

The statistical analysis was done by SPSS (IBM version 26). The categorical variables (demographic data, information about TM, knowledge about TM, attitude toward TM, and practice toward TM) were presented as frequencies and percentages. After recording, the correct answers to the knowledge questions were summed, resulting in a numerical score variable. Additionally, the questions of the attitude as well as the questions of the practice were summed, resulting in two numerical variables. Mann-Whitney and Kruskal-Wallis tests were used to compare the three outcome numerical variables and the demographic data to present medians, inter-quartile ranges, and P values. Generalized linear regression models were constructed to predict the high knowledge, attitude, and practice scores based on the statistically significant demographic data for each primary outcome. The regression analysis results were presented as beta coefficients and their respective 95% confidence intervals. A p-value of < 0.05 indicated statistical significance.

Ethical approval was sought from the University of Bisha Institutional Review Board (IRB) prior to initiating study activities. Participants were informed about the study's objectives, and individual consent was obtained before they filled out the questionnaires. Participants were also informed of their right to withdraw from the study at any time. Confidentiality was upheld throughout the study, ensuring that the respondents' information was kept private and secure.

## Results

Sociodemographic data of the participants

A total of 555 participants agreed to participate in the current study. The sociodemographic characteristics of the participants are presented in Table 1. The majority of participants were females (68.6%) and Saudi nationals (95.9%). Regarding age distribution, the largest proportion of participants fell within the age range of 18-25 (27.2%). In terms of educational level, the most common category was a bachelor's degree (56.6%), followed by a diploma degree (14.8%). The majority of participants were married (83.2%), and the most prevalent occupation was in the government sector (52.4%). Most participants reported a monthly income greater than 10,000 Saudi Riyals (42.5%), and the majority identified as non-smokers (87.0%). Additionally, 28.8% of participants reported that their occupation was related to healthcare.

**Table 1:** Sociodemographic data of the participants.

Parameter	Category	N	%
Age (years)	18-25	151	27.20%
	26-35	137	24.70%
	36-45	146	26.30%
	> 45	121	21.80%
Gender	Male	174	31.40%
	Female	381	68.60%
Nationality	Saudi	532	95.90%
	Non-Saudi	23	4.10%
Marital status	Married	462	83.20%
	Divorced	46	8.30%
	Widowed	47	8.50%
Educational level	illiterate	17	3.10%
	Elementary	14	2.50%
	Intermediate	17	3.10%
	Secondary	63	11.40%
	Diploma	82	14.80%
	Bachelor's degree	314	56.60%
	Higher education	48	8.60%
Occupation	Unemployment	169	30.60%
	Student	15	2.70%
	Private	73	13.20%
	Government	290	52.40%
	Retired	6	1.10%
Monthly income (Saudi Riyal)	3000-5000	195	35.10%
	5000-10000	124	22.30%
	> 10000	236	42.50%
Smoking status	I have never smoked	483	87.00%
	Previous smoker	41	7.40%
	Smoker	31	5.60%
Is your occupation related to healthcare?	No	395	71.20%
	Yes	160	28.80%

Patterns of use and knowledge regarding TM

**Figure 1:**
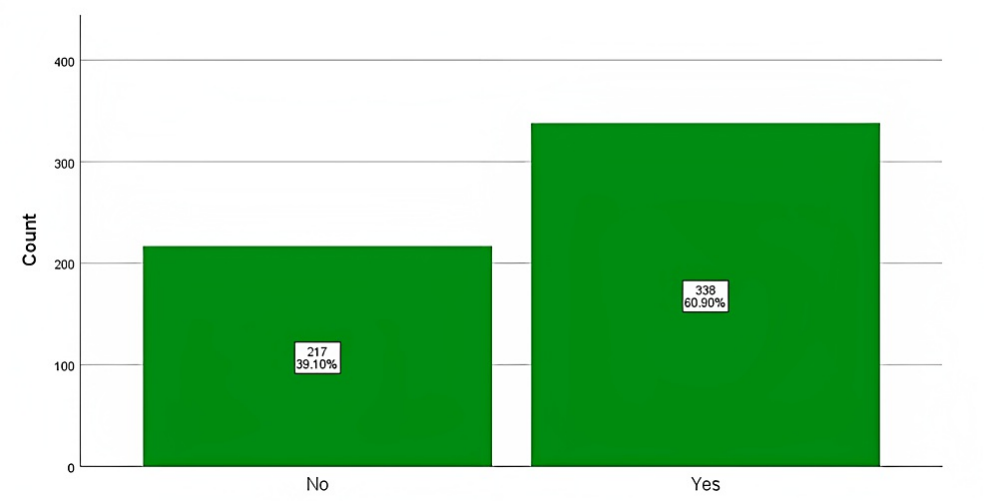
The proportion of participants who had ever tried traditional medicine and those who did not.

**Figure 2:**
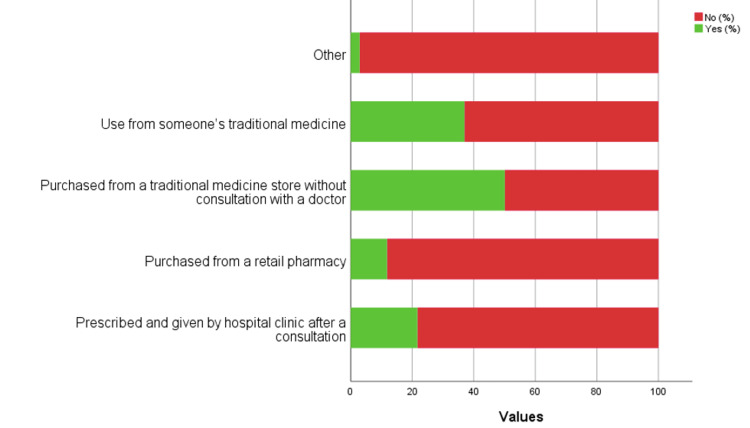
Sources of purchasing traditional medicine products.

**Figure 3:**
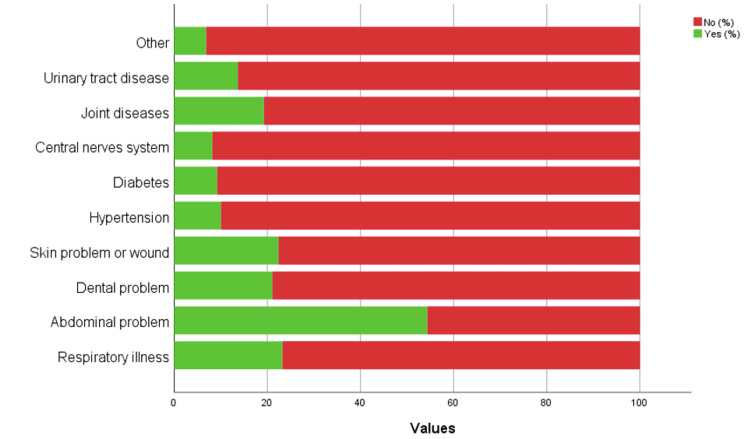
The reason for which participants had taken the traditional medicine products.

More than half of the participants have tried TM 338 (60.90%, Figure 1). Nearly half of the participants purchased from a TM store without consultation with a doctor (203, 50.10%; Figure 2) and tried TM for abdominal problems (206, 54.40%; Figure 3). Most of the participants disagreed that TM can prevent all diseases (317, 57.10%) or can cure all illnesses (323, 58.20%), while it was considered always safe by 301 (54.20%) and do not expire by 226 (40.70%). On the other hand, most of the participants accepted that TM is preferred because of fewer side effects (241, 43.40%) and can be taken with allopathic medicines (221, 39.80%). More than half of the participants accepted that TM is made from plant sources (430, 77.50%). Most of the participants were not sure if TM can be from an animal source or not (227, 40.90%).

Knowledge scores and demographic characteristics

The median (interquartile range, IQR) knowledge score was 4.0 (2.0-6.0). Table 2 presents the differences in knowledge scores based on various demographic characteristics. Participants aged > 45 demonstrated the highest median knowledge score (5.0), followed by those aged 26-35 and 36-45 (both with a median score of 4.0, p = 0.022). There were no significant differences in knowledge scores based on gender. In terms of marital status, married and widowed participants had the highest median knowledge score (4.0) compared to their divorced peers (3.0, p = 0.044). Educational level showed a significant association with knowledge scores, with illiterate participants having the lowest median score (2.0) and bachelor's degree holders having the highest median score (5.0, p < 0.001). There were no significant differences in knowledge scores based on smoking status or whether the occupation was related to healthcare (Table 2).

**Table 2:** The difference in knowledge scores based on demographic characteristics. IQR: interquartile range

Parameter	Category	Median (IQR)	P value
Age (years)	18-25	4.0 (2.0-5.0)	0.022
	26-35	4.0 (2.5-6.0)	
	36-45	4.0 (2.0-6.0)	
	> 45	5.0 (3.0-6.0)	
Gender	Male	4.0 (2.0-6.0)	0.810
	Female	4.0 (3.0-6.0)	
Nationality	Saudi	4.0 (2.25-6.0)	0.558
	Non-Saudi	4.0 (2.0-5.0)	
Marital status	Married	4.0 (3.0-6.0)	0.044
	Divorced	3.0 (2.0-6.0)	
	Widowed	4.0 (2.0-5.0)	
Educational level	illiterate	2.0 (1.0-3.0)	< 0.001
	Elementary	3.5 (1.0-5.0)	
	Intermediate	3.0 (1.5-3.0)	
	Secondary	4.0 (2.0-5.0)	
	Diploma	4.0 (2.0-5.0)	
	Bachelor's degree	5.0 (3.0-6.0)	
	Higher education	4.5 (2.0-6.0)	
Occupation	Unemployment	4.0 (3.0-5.0)	0.065
	Student	5.0 (2.0-6.0)	
	Private	3.0 (2.0-6.0)	
	Government	4.0 (3.0-6.0)	
	Retired	5.5 (4.75-7.5)	
Monthly income (Saudi Riyal)	3,000-5,000	4.0 (2.0-6.0)	0.043
	5,000-10,000	4.0 (2.0-5.0)	
	> 10,000	5.0 (3.0-6.0)	
Smoking status	I have never smoked	4.0 (3.0-6.0)	0.284
	Previous smoker	3.0 (2.0-5.0)	
	Smoker	5.0 (3.0-6.0)	
Is your occupation related to healthcare?	No	4.0 (3.0-6.0)	0.395
	Yes	4.0 (2.0-6.0)	

The multivariable regression analysis (Table 3) revealed several significant associations with high knowledge scores. Participants in the age groups 18-25 (beta = -0.774, 95% CI -1.310 to -0.237, p = 0.005), 26-35 (beta = -0.596, 95% CI -1.132 to -0.059, p = 0.030), and 36-45 (beta = -0.760, 95% CI -1.265 to -0.255, p = 0.003) showed significantly lower knowledge scores compared to those > 45 years (reference group). Lower educational levels, including illiterate (beta = -2.097, 95% CI -3.260 to -0.933, p < 0.001), elementary (beta = -1.614, 95% CI -2.879 to -0.348, p = 0.013), and intermediate (beta = -1.809, 95% CI -2.949 to -0.669, p = 0.002), were associated with lower knowledge scores compared to higher education (reference group). Other demographic variables did not show significant associations with high knowledge scores (Table 3).

**Table 3:** Results of the multivariable regression analysis for high knowledge scores.

Parameter	Category	Beta	95% CI		P value
			LB	UB	
Age (years)	18-25	-0.774	-1.310	-0.237	0.005
	26-35	-0.596	-1.132	-0.059	0.030
	36-45	-0.760	-1.265	-0.255	0.003
	> 45	Ref.	Ref.	Ref.	Ref.
Marital status	Married	0.18	-0.460	0.821	0.58
	Divorced	-0.338	-1.184	0.508	0.433
	Widowed	Ref.	Ref.	Ref.	Ref.
Educational level	illiterate	-2.097	-3.260	-0.933	< 0.001
	Elementary	-1.614	-2.879	-0.348	0.013
	Intermediate	-1.809	-2.949	-0.669	0.002
	Secondary	-0.520	-1.300	0.261	0.192
	Diploma	-0.832	-1.567	-0.098	0.026
	Bachelor's degree	0.053	-0.570	0.676	0.867
	Higher education	Ref.	Ref.	Ref.	Ref.
Monthly income (Saudi Riyal)	3,000-5,000	0.049	-0.391	0.489	0.826
	5,000-10,000	-0.225	-0.687	0.237	0.338
	> 10,000	Ref.	Ref.	Ref.	Ref.

Participants' attitudes toward TM use

**Figure 4:**
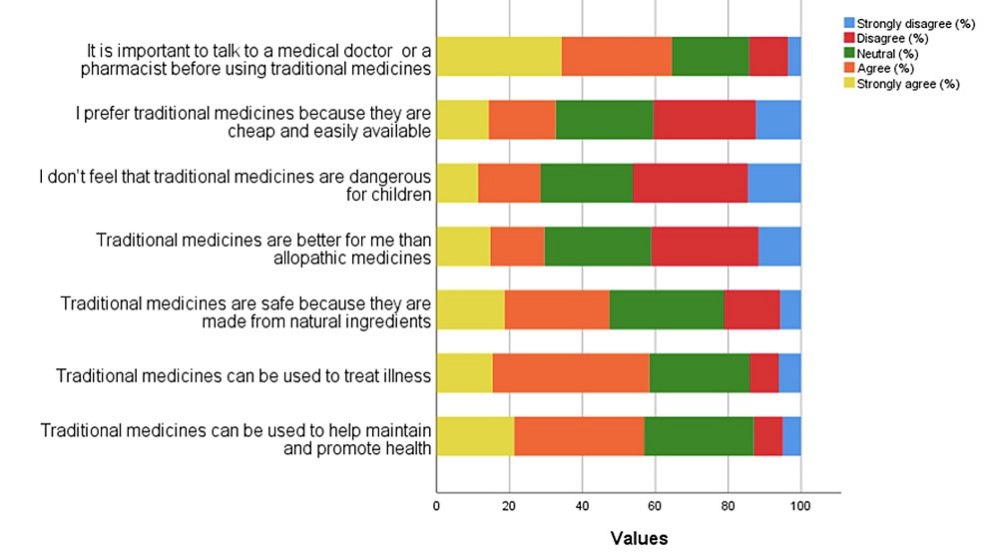
Participants’ responses to attitudes items.

Most of the participants agreed that TM can be used to help maintain and promote health (198, 35.70%) and treat illness (239, 43.10%). Most of the participants strongly agreed that it is important to talk to a medical doctor or pharmacist before using TM (190, 34.20%). Most of the participants disagreed that TM is cheap and easily available (156, 28.10%) and not dangerous for children (175, 31.50%). Most of the participants answered neutrally that TM is safe because it is made from natural ingredients (175, 31.50%; Figure 4).

Attitude scores and demographic characteristics

The median (IRQ) attitude score was 22.0 (20.0-26.0). Table 4 displays the differences in attitude scores based on various demographic characteristics. Participants in the age groups 18-25 and 26-35 years had the highest median attitude scores (23.0), followed by those aged 36-45 years (with a median score of 22.0, p = 0.001). Males demonstrated higher median attitude scores (24.0) compared to females (22.0, p = 0.028). Nationality, marital status, and monthly income did not show significant associations with attitude scores. However, educational level exhibited a significant association, with illiterate participants having the highest median attitude score (27.0, p = 0.008). Similarly, participants whose occupation was related to healthcare had significantly higher median attitude scores (23.0) compared to those whose occupation was not related to healthcare (22.0, p = 0.002).

**Table 4:** The difference in attitude scores based on demographic characteristics. IQR: interquartile range

Parameter	Category	Median (IQR)	P value
Age (years)	18-25	23.0 (21.0-28.0)	0.001
	26-35	23.0 (20.0-27.0)	
	36-45	22.0 (19.75-25.0)	
	> 45	21.0 (19.0-25.0)	
Gender	Male	24.0 (20.0-27.0)	0.028
	Female	22.0 (20.0-26.0)	
Nationality	Saudi	22.0 (20.0-26.0)	0.114
	Non-Saudi	24.0 (22.0-28.0)	
Marital status	Married	22.0 (19.75-26.0)	0.160
	Divorced	22.5 (20.0-26.0)	
	Widowed	24.0 (21.0-29.0)	
Educational level	illiterate	27.0 (24.0-35.0)	0.008
	Elementary	21.0 (16.75-24.25)	
	Intermediate	22.0 (21.0-24.5)	
	Secondary	24.0 (21.0-27.0)	
	Diploma	22.0 (20.0-27.0)	
	Bachelor's degree	22.0 (19.0-26.0)	
	Higher education	21.0 (19.0-28.0)	
Occupation	Unemployment	23.0 (20.0-26.0)	0.165
	Student	21.0 (18.0-22.0)	
	Private	23.0 (20.0-28.0)	
	Government	22.0 (19.0-26.0)	
	Retired	21.5 (17.5-27.0)	
Monthly income (Saudi Riyal)	3,000-5,000	23.0 (20.0-26.0)	0.202
	5,000-10,000	22.0 (20.0-26.75)	
	> 10,000	22.0 (19.0-26.0)	
Smoking status	I have never smoked	22.0 (20.0-26.0)	0.183
	Previous smoker	25.0 (20.0-28.0)	
	Smoker	24.0 (19.0-28.0)	
Is your occupation related to healthcare?	No	22.0 (19.0-26.0)	0.002
	Yes	23.0 (21.0-27.0)	

The multivariable regression analysis (Table 5) revealed significant associations with high attitude scores. Younger age groups, specifically 18-25 years (beta = 2.20, 95% CI 0.846-3.554, p = 0.001) and 26-35 years (beta = 1.407, 95% CI 0.036-2.778, p = 0.044), were associated with higher attitude scores compared to participants aged > 45 years (reference group). Males exhibited significantly higher attitude scores (beta = 1.208, 95% CI 0.222-2.193, p = 0.016) compared to females. Participants with lower educational levels, including illiterate (beta = 4.482, 95% CI 1.465-7.499, p = 0.004), showed significantly higher attitude scores compared to higher education (reference group). Additionally, participants whose occupation was not related to healthcare had significantly lower attitude scores (beta = -1.315, 95% CI -2.347 to -0.283, p = 0.013) compared to those whose occupation was related to healthcare.

**Table 5:** Results of the multivariable regression analysis for high attitude scores.

Parameter	Category	Beta	95% CI		P value
			LB	UB	
Age (years)	18-25	2.20	0.846	3.554	0.001
	26-35	1.407	0.036	2.778	0.044
	36-45	0.48	-0.855	1.814	0.48
	> 45	Ref.	Ref.	Ref.	Ref.
Gender	Male	1.208	0.222	2.193	0.016
	Female	Ref.	Ref.	Ref.	Ref.
Educational level	illiterate	4.482	1.465	7.499	0.004
	Elementary	-2.234	-5.512	1.044	0.181
	Intermediate	-0.157	-3.157	2.842	0.918
	Secondary	0.48	-1.564	2.524	0.645
	Diploma	0.375	-1.584	2.335	0.707
	Bachelor's degree	-0.514	-2.175	1.146	0.543
	Higher education	Ref.	Ref.	Ref.	Ref.
Is your occupation related to healthcare?	No	-1.315	-2.347	-0.283	0.013
	Yes	Ref.	Ref.	Ref.	Ref.

Participants’ practice of TM

**Figure 5:**
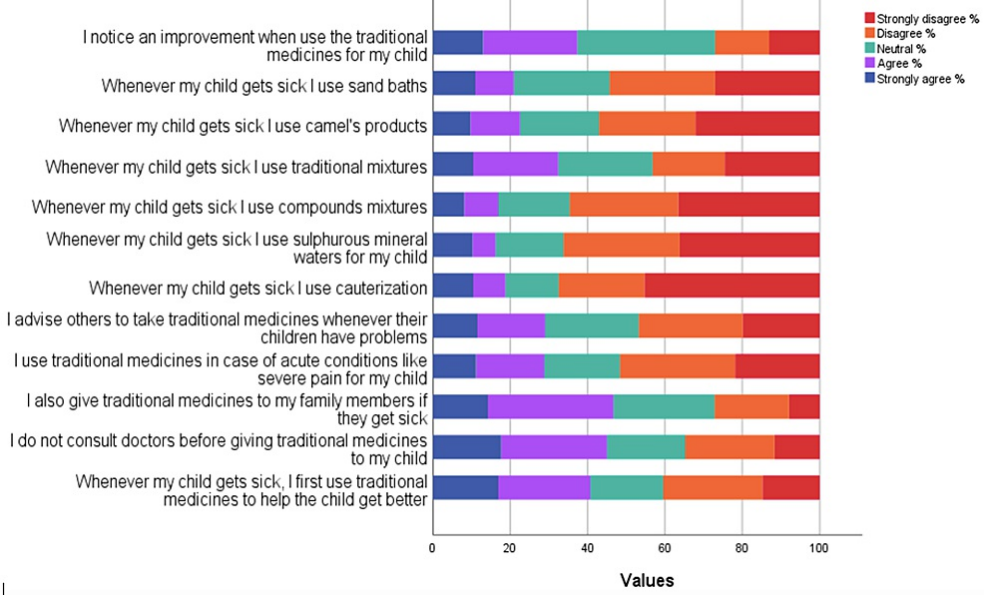
Participants’ responses to practice items.

The study results indicated that the median practice score was 31.0, with an IQ) of 24.0-38.0. Out of the surveyed participants, 32.40% (180 individuals) agreed that they would administer TM to their family members if they fell ill. However, a number of participants disagreed with certain practices related to TM. Specifically, 25.80% (143 individuals) disagreed with the idea of using TM as the primary treatment for a sick child. Similarly, 27.40% (152 individuals) stated they would not administer TM to their children without first consulting a doctor. In the case of acute conditions such as severe pain, 29.90% (166 individuals) disagreed with using TM as an immediate remedy for their children. Furthermore, 27.00% (150 individuals) indicated that they would not recommend others to use TM. Lastly, the same percentage (27.40% or 152 individuals) disagreed with using sand baths as a treatment method when their children were sick. Most of the participants strongly disagreed that they use cauterization whenever their children get sick (251, 45.20%), use sulfurous mineral waters whenever their children get sick (201, 36.20%), use compound mixtures whenever their children get sick (203, 36.60%), and use camel products whenever their children get sick (178, 32.10%). Most of the participants answered neutrally that they noticed an improvement when they use the TM for their children (198, 35.70%; Figure 5).

Practice scores based on demographic characteristics

Table 6 illustrates the differences in practice scores based on various demographic characteristics. Participants in the age group 18-25 had the highest median practice score (36.0), followed by those aged 26-35 (31.0, p < 0.001). Males demonstrated higher median practice scores (34.0) compared to females (31.0, p = 0.003). Saudi participants had a lower median practice score (31.0) compared to non-Saudi participants (38.0, p = 0.045). Marital status and monthly income did not show significant associations with practice scores. However, educational level exhibited a significant association, with illiterate participants having the highest median practice score (38.0, p = 0.003). Similarly, participants whose occupation was related to healthcare had significantly higher median practice scores (35.0) compared to those whose occupation was not related to healthcare (31.0, p = 0.002).

**Table 6:** The difference in practice scores based on demographic characteristics.

Parameter	Category	Median (IQR)	P value
Age (years)	18-25	36.0 (29.0-42.0)	< 0.001
	26-35	31.0 (22.5-40.0)	
	36-45	31.0 (26.0-37.0)	
	> 45	28.0 (22.0-34.0)	
Gender	Male	34.0 (26.0-42.25)	0.003
	Female	31.0 (23.0-37.0)	
Nationality	Saudi	31.0 (24.0-38.0)	0.045
	Non-Saudi	38.0 (29.0-43.0)	
Marital status	Married	31.0 (23.75-38.0)	0.032
	Divorced	34.5 (24.75-38.25)	
	Widowed	36.0 (29.0-41.0)	
Educational level	illiterate	38.0 (32.5-56.0)	0.003
	Elementary	30.5 (26.0-34.5)	
	Intermediate	35.0 (27.0-39.0)	
	Secondary	34.0 (28.0-43.0)	
	Diploma	31.0 (25.0-40.0)	
	Bachelor's degree	31.0 (23.0-37.0)	
	Higher education	31.0 (23.25-36.75)	
Occupation	Unemployment	31.0 (25.0-37.0)	0.287
	Student	28.0 (20.0-36.0)	
	Private	34.0 (25.5-40.5)	
	Government	31.0 (23.0-38.0)	
	Retired	28.0 (23.5-31.5)	
Monthly income (Saudi Riyal)	3,000-5,000	32.0 (26.0-38.0)	0.295
	5,000-10,000	32.0 (25.0-38.75)	
	> 10,000	31.0 (23.0-38.0)	
Smoking status	I have never smoked	31.0 (24.0-38.0)	0.216
	Previous smoker	33.0 (26.0-40.0)	
	Smoker	33.0 (28.0-43.0)	
Is your occupation related to healthcare?	No	31.0 (24.0-36.0)	0.002
	Yes	35.0 (26.0-42.0)	

The multivariable regression analysis (Table 7) revealed significant associations with high practice scores. Younger age groups, particularly 18-25 years (beta = 6.821, 95% CI 4.380-9.262, p < 0.001) and 26-35 years (beta = 4.028, 95% CI 1.569-6.487, p = 0.001) and 36-45 years (beta = 3.392, 95% CI 1.008-5.776, p = 0.005), were independently associated with higher practice scores compared to participants aged > 45 years (reference group). Males exhibited independently significantly higher practice scores (beta = 3.623, 95% CI 1.856-5.391, p < 0.001) compared to females. Illiterate participants showed significantly higher practice scores (beta = 9.205, 95% CI 3.734-14.676, p = 0.001) compared to higher education (reference group). Furthermore, participants whose occupation was not related to healthcare had significantly lower practice scores (beta = -1.852, 95% CI -3.701 to -0.003, p = 0.05) compared to those whose occupation was related to healthcare. 

**Table 7:** Results of the multivariable regression analysis for high practice scores.

Parameter	Category	Beta	95% CI		P value
			LB	UB	
Age (years)	18-25	6.821	4.380	9.262	< 0.001
	26-35	4.028	1.569	6.487	0.001
	36-45	3.392	1.008	5.776	0.005
	> 45	Ref.	Ref.	Ref.	Ref.
Gender	Male	3.623	1.856	5.391	< 0.001
	Female	Ref.	Ref.	Ref.	Ref.
Nationality	Saudi	-1.892	-5.950	2.166	0.36
	Non-Saudi	Ref.	Ref.	Ref.	Ref.
Marital status	Married	-1.688	-4.728	1.351	0.276
	Divorced	-1.436	-5.469	2.597	0.485
	Widowed	Ref.	Ref.	Ref.	Ref.
Educational level	illiterate	9.205	3.734	14.676	0.001
	Elementary	0.653	-5.258	6.564	0.828
	Intermediate	1.844	-3.518	7.205	0.50
	Secondary	2.788	-0.866	6.441	0.134
	Diploma	2.422	-1.074	5.917	0.174
	Bachelor's degree	0.122	-2.842	3.086	0.936
	Higher education	Ref.	Ref.	Ref.	Ref.
Is your occupation related to healthcare?	No	-1.852	-3.701	-0.003	0.05
	Yes	Ref.	Ref.	Ref.	Ref.

## Discussion

Our study aimed to explore the patterns of use, knowledge, attitudes, and practices regarding TM among a sample of 555 participants. The sociodemographic characteristics of the participants indicated a predominantly female population, with a high percentage of Saudi nationals. The majority of participants fell within the age range of 18-25, held a bachelor’s degree, were married, and worked in the government sector. These sociodemographic factors provide insights into the population under study and help contextualize the findings.

Our study investigated the patterns of use and knowledge regarding TM among the participants. The findings revealed that more than half of the participants had tried TM, indicating its popularity and widespread usage in the studied population. This finding is consistent with previous studies that have reported high rates of TM utilization [10,11]. The availability and accessibility of TM stores may contribute to this prevalence, as nearly half of the participants reported purchasing TM without consulting a doctor. This highlights the need for better regulation and supervision of TM outlets to ensure the safety and efficacy of the products.

One of the common reasons reported by the participants for using TM was its perceived ability to alleviate abdominal problems. This aligns with previous studies that have identified gastrointestinal disorders as one of the primary conditions for which TM is sought [12,13]. However, it is important to note that self-medication without medical consultation may not always be appropriate, as it can lead to potential risks and delays in seeking proper medical care. Healthcare providers should be aware of this trend and engage in open communication with patients regarding their use of TM.

Participants' knowledge and beliefs regarding TM varied in certain aspects. While a majority of participants did not believe that TM can prevent or cure all diseases, they still acknowledged its benefits, such as fewer side effects and compatibility with allopathic medicines. These findings are consistent with studies that have highlighted the perception of TM as a safer alternative to conventional medicine due to its natural origins and potentially milder side effects [14].

Interestingly, although most participants recognized that TM is predominantly derived from plant sources, there was uncertainty regarding the use of animal-based ingredients in TM. This knowledge gap suggests a need for public education and awareness campaigns to enhance understanding of the composition and sources of TM. It is important for individuals to be aware of any potential allergens or contraindications associated with specific TM ingredients, including those derived from animal sources.

The analysis of knowledge scores based on demographic characteristics revealed several noteworthy findings. Age was found to be significantly associated with knowledge scores, with participants aged > 45 demonstrating the highest median score. This result may be attributed to accumulated knowledge and experience with TM over time. In contrast, younger age groups (18-25, 26-35, and 36-45) had lower knowledge scores, indicating a potential generational gap in understanding TM. Similar age-related differences in knowledge have been reported in previous studies [15].

The educational level also showed a significant association with knowledge scores, with higher levels of education being positively correlated with greater knowledge. Illiterate individuals had the lowest median scores, while those with a bachelor’s degree had the highest. This finding is consistent with previous research, which has consistently shown that higher education levels are associated with better health literacy and knowledge about healthcare practices [16].

The present study assessed participants’ attitudes toward TM use and explored the associations between attitude scores and demographic characteristics. The findings revealed the majority of participants agreed that TM could be utilized to maintain and promote health as well as treat illnesses. This aligns with the growing recognition of TM as a complementary approach to healthcare [17]. It is noteworthy that most participants strongly believed in the importance of consulting medical professionals before using TM, highlighting their awareness of potential risks and the need for expert advice [18].

Contrary to the participants’ agreement on the efficacy of TM, a significant proportion disagreed that it is cheap, easily available, and safe for children. This finding suggests a potential misconception or lack of awareness regarding the accessibility and safety aspects of TM, which requires further attention in public health education and awareness campaigns [19].

The analysis of attitude scores in relation to demographic characteristics revealed several noteworthy associations. Firstly, younger participants, particularly those in the age groups of 18-25 and 26-35 years, exhibited higher attitude scores, suggesting a generational shift in attitudes toward TM. This aligns with previous studies reporting a positive attitude toward TM among younger populations [20]. Gender was also found to be associated with attitude scores, with males demonstrating higher scores compared to females, possibly reflecting variations in cultural beliefs and gender roles [21]. Educational level emerged as a significant factor, with illiterate participants exhibiting the highest attitude scores, while individuals with higher education showed greater skepticism toward TM [22,23]. Additionally, healthcare-related occupations were associated with higher attitude scores, indicating better understanding and acceptance of TM's benefits among healthcare workers [24]. This emphasizes the role of healthcare providers in educating the public about TM use.

Regarding the practice of TM among participants and the associations between demographic characteristics and practice scores, the findings revealed that the median practice score was 31.0, indicating a moderate level of engagement in TM practices. One notable aspect of the participants’ practice was their agreement that TM can be given to family members if they become ill. This finding is consistent with previous studies that have reported a positive attitude toward using TM within the family context [25]. However, it is worth noting that a considerable proportion of participants disagreed with the idea of using TM as the first line of treatment for their children, indicating a preference for seeking medical advice from doctors. This finding aligns with the study by Astin et al., which highlighted the importance of seeking professional medical care before resorting to traditional remedies [26].

Furthermore, participants strongly disagreed with the use of certain traditional practices such as cauterization, sulfurous mineral waters, compound mixtures, and camel products when their children fell ill. This reluctance to engage in potentially harmful practices reflects an increasing awareness among the participants about the potential risks associated with certain traditional healing modalities. Similar findings have been reported in other studies, indicating a shift toward more cautious attitudes regarding harmful traditional practices [27].

When analyzing the practice scores based on demographic characteristics, several significant associations were observed. Age was found to be a determining factor, with younger participants (18-25 years, 26-35 years, and 36-45 years) exhibiting higher practice scores compared to those aged over 45 years. This finding suggests that younger individuals may be more inclined to embrace TM practices compared to older generations. This result is consistent with studies that reported similar age-related patterns in TM usage [28].

Gender also emerged as a significant factor, with males demonstrating higher practice scores than females. This finding may be attributed to cultural and societal factors influencing the gender-specific adoption of TM practices. Previous studies have also identified gender disparities in TM usage, with men often being more receptive to such practices [29].

Interestingly, participants with a lower educational level, particularly illiterate individuals, exhibited higher practice scores compared to those with higher education. This finding is intriguing and warrants further exploration. One possible explanation is that individuals with lower levels of education may have limited access to formal healthcare services, leading them to rely more heavily on TM practices as an alternative form of treatment. This finding is consistent with studies that reported similar associations between education level and TM usage [30].

Occupation also played a significant role in participants’ practice scores. Those whose occupation was related to healthcare exhibited higher practice scores compared to those in non-healthcare-related occupations. This finding implies that individuals with a professional background in healthcare may have a better understanding of the benefits and limitations of TM, leading them to integrate these practices into their personal healthcare choices. This result aligns with studies that emphasized the influence of occupation on TM utilization [31].

It is important to acknowledge that this study has certain limitations. Firstly, the data collected relied on self-reporting, which may be subject to recall bias or social desirability bias. Secondly, the study was conducted within a specific population and geographical context, limiting the generalizability of the findings. Future research could explore similar topics in diverse populations to gain a more comprehensive understanding of TM utilization.

## Conclusions

The study found that over half of the participants had used TM, with many purchasing it without consulting a doctor. Participants had mixed attitudes toward TM, with most agreeing that it can be used to treat illness and promote health, but disagreeing that it is cheap and easily available or safe for children. The study also found that demographic factors, such as age and educational level, were significantly associated with knowledge, attitude, and practice scores related to TM. Further research is warranted to explore the underlying factors contributing to these differences.
